# Silver and Copper Nanoparticles Hosted by Carboxymethyl Cellulose Reduce the Infective Effects of Enterotoxigenic *Escherichia coli*:F4 on Porcine Intestinal Enterocyte IPEC-J2

**DOI:** 10.3390/microorganisms12102026

**Published:** 2024-10-07

**Authors:** Armelle Tchoumi Neree, Farzaneh Noori, Abdelkrim Azzouz, Marcio Costa, John Morris Fairbrother, Mircea Alexandru Mateescu, Younes Chorfi

**Affiliations:** 1 Department of Veterinary Biomedical Sciences, Faculty of Veterinary Medicine, Université de Montréal, St-Hyacinthe, QC J2S 2M2, Canada; marcio.costa@umontreal.ca (M.C.); younes.chorfi@umontreal.ca (Y.C.); 2 Swine and Poultry Infectious Research Center (CRIPA-FRQNT), Faculty of Veterinary Medicine, Université de Montréal, St-Hyacinthe, QC J2S 2M2, Canada; john.morris.fairbrother@umontreal.ca; 3 Department of Chemistry, Université du Québec à Montréal, Montréal, QC H3C 3P8, Canada; noorifarzaneh2@gmail.com (F.N.); azzouz.a@uqam.ca (A.A.); 4 World Organisation for Animal Health (OIE), Reference Laboratory for *Escherichia coli* (EcL), Faculty of Veterinary Medicine, Université de Montréal, St-Hyacinthe, QC J2S 2M2, Canada

**Keywords:** enterotoxigenic *Escherichia coli* fimbriae 4 (ETEC:F4), carboxymethyl cellulose (CMC), copper nanoparticles (Cu^0^NP), intestinal porcine enterocytes (IPEC-J2), silver nanoparticles (Ag^0^NP)

## Abstract

Zero-valent copper and silver metals (Ms) nanoparticles (NPs) supported on carboxymethylcellulose (CMC) were synthesized for treating Enterotoxigenic *Escherichia coli* fimbriae 4 (ETEC:F4), a major cause of diarrhea in post-weaned pigs. The antibacterial properties of Cu^0^/CMC and Ag^0^/CMC were assessed on infected porcine intestinal enterocyte IPEC-J2, an in vitro model mimicking the small intestine. The lower average particle size (218 nm) and polydispersity index [PDI]: 0.25) for Ag^0^/CMC, when compared with those of Cu^0^/CMC (367 nm and PDI 0.96), were explained by stronger Ag^0^/CMC interactions. The minimal inhibitory concentration (MIC) and half inhibitory concentration (IC_50_) of Ag^0^/CMC were lower in both bacteria and IPEC-J2 cells than those of Cu^0^/CMC, confirming that silver nanoparticles are more bactericidal than copper counterparts. IPEC-J2, less sensitive in MNP/CMC treatment, was used to further investigate the infective process by ETEC:F4. The IC_50_ of MNP/CMC increased significantly when infected IPEC-J2 cells and ETEC were co-treated, showing an inhibition of the cytotoxicity effect of ETEC:F4 infection and protection of treated IPEC-J2. Thus, it appears that metal insertion in CMC induces an inhibiting effect on ETEC:F4 growth and that MNP/CMC dispersion governs the enhancement of this effect. These results open promising prospects for metal-loaded biopolymers for preventing and treating swine diarrhea.

## 1. Introduction

Enterotoxigenic *Escherichia coli* (ETEC) are pathogenic bacteria which adhere to the microvilli of small intestinal epithelial cells and colonize the gut [1,2]. Diarrhea in swine is most associated with ETEC possessing fimbriae 4 (ETEC:F4) [3]. The pathogenicity of ETEC:F4 strains is due to the adhesive properties of fimbriae 4 (F4) and the enterotoxins released by attached bacteria. These factors are responsible for severe watery diarrhea and weight loss [4,5].

Antimicrobial resistance has emerged as a cause of economic losses in pig production and as a threat to the human healthcare system, caused by the misuse and overuse of antibiotics. More than 90% of the ETEC:F4 isolates in pigs may be multidrug-resistant [6]. Novel and alternative treatments are now targeted to address this issue. Among the approaches tackled so far, the use of the antimicrobial properties of metal nanoparticles (MNPs) is of great interest. The benefit effects of MNPs are related to their ability to induce bacterial membrane damage, bacteria growth inhibition [7], and death [8]. Moreover, the free radicals, resulting from the action of metals, may damage the bacterial DNA [9,10]. We hypothesized that CMC-hosted Ag^0^NP and Cu^0^NP could mitigate ETEC:F4 infection in porcine intestinal enterocytes IPEC-J2. Silver has been known for centuries as an antiseptic material with relatively low toxicity. In addition, to freely penetrate cellular barriers and generate damaging reactive oxygen species (ROS), nanoparticles of silver ions are also able to intercalate into nucleic acids, inhibiting the transcription and translation processes [11]. Also, copper NPs (Cu NPs) show good antimicrobial action [12,13], with a high capacity to generate ROS and at a lower cost, compared with silver.

Unlike metals in compact form, dispersed metal particles exhibit high surface-to-bulk ratios and improved contact surfaces that promote their biocidal effects [14]. Metal dispersion and stabilization is a major challenge that requires efficient ion or metal trapping materials. Natural and plant-deriving carbohydrate polymers such as cellulose or starch functionalized with anionic groups such as carboxylic groups offer promising prospects in this regard. For instance, carbohydrate-supported copper or silver nanoparticles (Cu^0^NP or Ag^0^NP) turned out to be effective for this purpose in concentration ranges that prevent toxicity for mammalian cells [11,15].

Carboxymethyl cellulose (CMC) is a commercial carbohydrate resulting from hydroxyl substitution in cellulose by a carboxymethyl group [16,17]. This modification confers to CMC as excipient gastric acid resistance by protonation of carboxylic groups and with the intestinal transit will be deprotonated and ionized, favoring intestinal drug delivery from oral dosage forms [18,19]. CMC is a low-cost commercially available biopolymer with good biodegradability, known as a generally recognized as safe (GRAS) compound, and it was employed herein as a matrix for MNPs. The CMC was also the first cellulose derivative approved by the Food and Drug Administration (FDA) [20].

Previous studies indicated that the Ag^+^ cation released from Ag^0^NP was responsible for the antibacterial activity [21]. Noori et al. [22] stated that the biocidal effect of metal-loaded carboxylated biopolymers is reversely proportional to their retention strength. This report presents silver and copper nanoparticles hosted by CMC with an investigation of antibacterial activity related to the structural parameters of the links of MNPs:CMC hosting material. The novelty is the observation of a markedly higher toxicity on *E. coli* bacteria than on epithelial IPEC-J2 cells as a model for the intestinal wall. A special attention will be paid to the inhibition of biofilm formation, related to microbial invasion, adhesion, and growth. All bactericidal aspects of MNP/CMC were compared with those of currently used antibiotics such as fosfomycin and kanamycin. The results are expected to allow for the correlation of the material properties to their efficiency as antibacterial agents.

## 2. Materials and Methods

### 2.1. Materials

Copper II acetate [Cu(CH_3_COO)_2_], silver nitrate (AgNO_3_), and sodium borohydride (NaBH_4_ 98%) were supplied by Fisher chemicals (Canada). Agar, carboxymethyl cellulose (CMC) with a degree of substitution (DS) of 0.92 ± 0.01 carboxymethyl group (CM) per glucose unit (Glc) and 90 kDa (MW), crystal violet, dexamethasone, 3-(4,5-dimethylthiazol-2-yl)-2,5-diphenyl-2H-tetrazolium bromide (MTT), epidermal growth factor (EGF), and Luria–Bertani broth (LB) were purchased from Sigma-Aldrich (Oakville, ON, Canada). A lactate dehydrogenase (LDH) assay kit was obtained from Promega (Madison, WI, USA). Dulbecco’s modified Eagle medium and Ham’s F-12 nutrient mixture (DMEM/F12 medium), fetal bovine serum (FBS), insulin/transferrin/selenium (ITS), penicillin, streptomycin, and trypsin were obtained from Wisent (Saint-Jean-Baptiste, QC, Canada). Intestinal porcine jejunal epithelial cell line (IPEC-J2) was purchased from the American Type Culture Collection (ATCC). Enterotoxigenic *Escherichia coli fimbriae* 4 (ETEC:F4), also called the EcL8559 strain, was kindly provided by the Reference Laboratory for *Escherichia coli* (EcL, Faculty of Veterinary Medicine, Université de Montréal). All current chemicals were of reagent grade and used without further purification.

### 2.2. Preparation of Cu^0^/CMC and Ag^0^/CMC

Metal-hosted carboxymethyl cellulose (CMC) was prepared according to Noori et al. [20] Cation-loaded CMC samples (Cu^2+^/CMC and Ag^+^/CMC) were prepared by adding dropwise 10 mL of 0.1 mol/L Cu(CH_3_COO)_2_ or AgNO_3_ solutions in 2% CMC solution (1 g of CMC in 50 mL of water) at 50–60 °C under stirring for 3 h. The reduction of the metal cations into zero-valent metals was carried out by introducing 4 mL of 0.5 M NaBH_4_ under a nitrogen stream for 10 min to obtain metal zero-hosted CMC samples (Cu^0^/CMC and Ag^0^/CMC). Both mixtures were then sonicated for 50 min (500 W, 20 kHz) at room temperature for homogenization. The resulting materials were dried by lyophilization and stored in an airtight container attached to a vacuum pump to prevent further oxidation of the obtained zero-valent metal nanoparticles loaded on CMC (MNP/CMC).

### 2.3. Characterization of MNP/CMC by Dynamic Light Scattering (DLS)

Dynamic light scattering (DLS) measurements were performed with a Malvern Zetasizer Nano ZS (Malvern, Herrenberg, Germany) equipped with a 633 nm He-Ne laser and operating at an angle of 173°. The analysis of the data was performed by Dispersion Technology Software version 6.01 from Malvern Panalytical Ltd. (Grovewood Road, Malvern, UK). A total volume of 2 mL of each sample at a concentration of 1 mg/mL was measured in single-use polystyrene cuvettes with a pathlength of 10 mm. The measurements were performed at a position of 4.65 mm from the cuvette wall with an automatic attenuator and at a controlled temperature of 25 °C. For each sample, 15 runs of 10 s were performed, with three repetitions. The distribution size, the Z-average diameter (Z-average), and the polydispersity index (PDI) were recorded.

### 2.4. Study of MNP/CMC by ATR-FTIR Spectroscopy

Structural aspects of the metal-based nanostructures deposited on a zinc selenide crystal surface was investigated by infrared spectroscopy using a single-bounce attenuated total reflection Fourier transform infrared (ATR-FTIR) spectrometer (PerkinElmer Spectrum 400, FTIR PerkinElmer Inc., Waltham, MA, USA). The samples were prepared using 1 mg of Cu^0^/CMC and 1 mg of Ag^0^/CMC and placed on the sample holder, and the ATR-FTIR spectra were acquired over a wavenumber range of 500–4000 cm^−1^ with a resolution of 4 cm^−1^. The spectra were normalized, and the baseline was corrected using Spectrum™ software (6.3.4, PerkinElmer, Waltham, MA, USA).

### 2.5. Enterotoxigenic E. coli F4 Fimbriae (ETEC:F4) Bacteria Culture

The enterotoxigenic *E. coli* F4 fimbriae (ETEC:F4) used in the present study was strain EcL8559, obtained from the EcL Laboratory Faculty of Veterinary Medicine, Université de Montréal). The experiments were conducted following biosafety level 2. Bacteria were cultured for 24 h (100 rpm, 37 °C) in Luria–Bertani broth (LB) and quantified from the absorbance at 600 nm. Colony-forming units of serial diluted fractions of bacteria were determined, and a fixed concentration of 1 × 10^7^ CFU/mL was used for further experimentations.

### 2.6. Porcine Intestinal Enterocyte IPEC-J2 Culture

The IPEC-J2 cells were seeded in a 25 cm^2^ flask and cultured at 37 °C, 5% CO_2_ in a humidified atmosphere in DMEM/F12 media supplemented with 10% fetal bovine serum (FBS), 1% insulin/transferrin/selenium (ITS), 5 μg/mL EGF, 100 units/mL penicillin, and 100 µg/mL streptomycin. According to ATCC guidelines, the cells were checked microscopically daily, and the medium was changed every two days until they reached 80% confluency. Then, the cells were detached with 0.25% trypsin-EDTA solution and split 1 in 10 for new seedlings in a 25 cm^2^ flask containing supplemented medium.

For cell viability experiments, the IPEC-J2 cells were seeded at a final concentration of 0.2 × 10^4^ cells/mL in 96-well microplates with a flat bottom (Cytiva, BC, Canada) and were incubated in a humidified atmosphere at 37 °C and 5% CO_2_ until confluency. Then, they were differentiated for 10 days in FBS-free medium, supplemented with 100 units/mL penicillin, 100 µg/mL streptomycin, and 5 μg/mL EGF. Differentiated cells were used for viability and for infection assays.

### 2.7. Bactericidal Activity of MNP/CMC

A final volume of 10 mL containing 1×10^7^ CFU/mL of ETEC:F4 EcL8559 was treated with 0–0.20 mg/mL of Cu^0^/CMC or Ag^0^/CMC for 24 h at 37 °C, 100 rpm. The optical density (OD) was measured at 600 nm using a spectrophotometer (SpectraMax M3, Molecular Devices, Sunnyvale, CA, USA). In addition, approximately 1×10^6^ CFU of ETEC:F4 was inoculated and spread on Petri dishes with agar gel prepared from a sterilized solution containing LB 25 g/L and agar 15 g/mL antibacterial agents (kanamycin and fosfomycin) or zero-valent metals loaded on CMC such as Cu^0^/CMC or Ag^0^/CMC, which were placed on the inoculated surface and incubated for 24 h at 37 °C. The CMC was used as negative control, whereas the positives controls were kanamycin and fosfomycin, usually used in human health and in the veterinary field, respectively. The blank represented by the LB medium was subtracted from the obtained data, and the non-treated bacteria represented 100% bacteria viability.

### 2.8. Disk Diffusion Assay

The method was adapted from that described in El-Riz et al. [23]. A volume of 100 μL of ETEC:F4 10^7^ CFU/mL was inoculated on LB agar dishes. Subsequently, a sterile disc was centered into inoculated LB agar dishes and was moistened with 10 μL of sterile LB. Around 1 mg of a sample of MNP/CMC was deposited on a disc, previously centered on agar gel. For the positive controls of bactericidal activity, kanamycin and fosfomycin antibiotics were used. All of the Petri dishes were incubated overnight at 37 °C. Finally, the antimicrobial activities of MNP/CMC were evaluated by measuring the diameter of inhibition in millimeters (mm).

### 2.9. Cytotoxic Effect of MNP/CMC on IPEC-J2

The stock solution of 1 mg/mL CMC (control) or MNP/CMC was sterilized with a 0.2 mm filter, and various concentrations of Cu^0^/CMC, Ag^0^/CMC, or CMC (0–20 mg/mL) were prepared in order to treat differentiated IPEC-J2 cells. Cytotoxicity effects were followed using the 3-(4,5-dimethylthiazol-2-yl)-2,5-diphenyl-2H-tetrazolium bromide (MTT) assay. After 24 h of treatment, cells were washed three times and treated with MTT (5 mg/mL phosphate buffered saline) for 4 h at 37 °C, 5% CO_2_. The MTT reduction by living cells into solid purple formazan is quantifiable via the optical density (OD) at 560 nm. The formazan crystals were solubilized by adding an equal volume of 10 mM SDS containing 10 mM HCl. The percent of survived cells was compared with that of the control, and the concentration of sample required to kill 50% of cells (IC_50_) was evaluated. The average of three wells was used to determine the mean of each point for three different experiments.

### 2.10. Infection Assay and Cytotoxicity Assay of MNP/CMC on Infected IPEC-J2

Prior to the infection process, ETEC:F4 bacteria were diluted to 1 × 10^7^ CFU/mL in 6-well plates; then washed three times with 1 × phosphate buffer saline (PBS) containing, in mM, NaCl (137), KCl (2.7), Na_2_HPO_4_ (10), and KH_2_PO_4_ (1.8) at pH 7.4; and finally resuspended in antibiotic-free medium supplemented with 1% fetal bovine serum (FBS). During the infection step, a total volume of 1 mL of ETEC:F4 was added to IPEC-J2 cells adhered in a well of a 6-well plate for an approximative multiplicity of infection (MOI) with a 10:1 ratio of deposited infectious bacteria divided by the number of target cells in the well. Plates were incubated for 24 h or for 48 h at 37 °C and 5% CO_2_.

For a complete investigation of effect of MNP/CMC against infection of IPEC-J2 by ETEC:F4, three situations were proposed.

Situation 1 [{IPEC-J2 cells + ETEC:F4} + MNP/CMC]: The IPEC-J2 cells were first infected with ETEC:F4 (MOI 10:1) for 24 h and then treated with 0–0.05 mg/mL of MNP/CMC for 24 h and 48 h.

Situation 2 [{IPEC-J2 cells + MNP/CMC} + ETEC:F4]: An amount of 10^6^ cells by well of differentiated IPEC-J2 were first treated with 0–0.05 mg/mL of MNP/CMC for 24 h or 48 h and then infected with 10^7^ CFU of ETEC:F4 (MOI 10:1) for 24 h.

Situation 3 [IPEC-J2 + {ETEC:F4 + MNP/CMC}]: The differentiated IPEC-J2 cells were infected with a suspension containing ETEC:F4 already treated with MNP/CMC. A volume of 1 mL of ETEC:F4 bacteria 10^7^ CFU/mL was incubated with 0–0.05 mg/mL of MNP/CMC for 24 h. The mixture obtained was added into differentiated IPEC-J2 and then incubated at 37 °C, 5% CO_2_ for 24 h or 48 h.

In all three situations, cells were cultured in antibiotic-free medium to avoid interferences. At the indicated time, the medium was collected and centrifuged at 800× *g* for 15 min at 4 °C, and the viability of IPEC-J2 was assayed via a lactate dehydrogenase (LDH) detection kit (Promega, Madison, WI, USA) according to the manufacturer guidelines. Compared with the MTT assay, the LDH assay provides a better reproducibility with infected IPEC-J2.

### 2.11. Statistical Analysis

All experiments used a minimum of three replicates (n = 3). Where relevant, data are expressed as the mean ± SD. Statistical tests were performed with GraphPad software using a one-way ANOVA test. Differences were deemed statistically significant when the associated *p*-value was less than 0.05.

Antibacterial effectiveness, half-maximal effective concentration (IC_50_), the concentration of Cu^0^/CMC and of Ag^0^/CMC that induced a response halfway between the baseline and maximum after a specified exposure time), and the minimal inhibitory concentration (MIC, the lowest concentrations of Cu^0^/CMC and Ag^0^/CMC that will inhibit the visible growth of the bacteria) were calculated in order to evaluate Cu^0^/CMC and Ag^0^/CMC antibacterial activity.

## 3. Results and Discussion

### 3.1. Dispersion of Cu^0^/CMC and of Ag^0^/CMC

The copper nanoparticles (Cu^0^/CMC) and silver nanoparticles (Ag^0^/CMC) were previously prepared through metal cation reduction into zero-valent metal on the polymeric support, according to Noori et al. [22].

**Figure 1:**
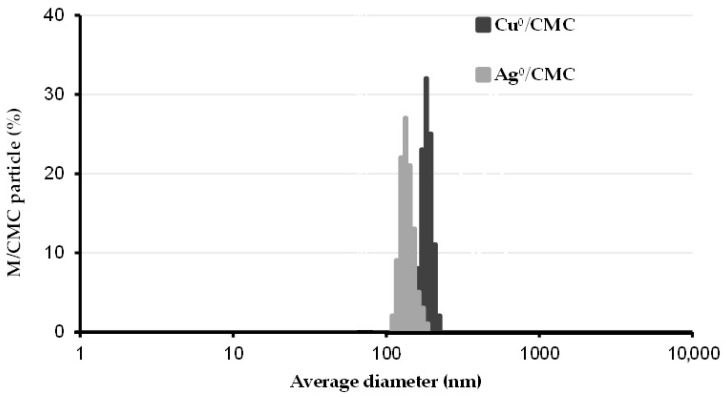
Particle size distribution of metal nanoparticle hosted by carboxymethyl cellulose (MNP/CMC). The size distribution of copper and silver nanoparticles hosted by carboxymethyl cellulose (Cu^0^/CMC and Ag^0^/CMC) was assessed by dynamic light scattering (DLS) in aqueous suspensions. Triplicate measurements were performed by DLS in 2 mL of polystyrene cell with a 10 mm pathlength.

The distribution of the particle size (PS) of the as-synthesized MNP/CMC was assessed through dynamic light scattering spectroscopy (DLS) in an aqueous suspension of 0.2 mg/mL phosphate-buffered saline (PBS). Cu^0^/CMC and Ag^0^/CMC showed a nanoparticle size maximal value at 210 ± 11 nm and 368.6 ± 23 nm (Figure 1). These values agree with the corresponding z-average values (367 ± 39 nm and 218 ± 17 nm, respectively) (Table 1). Here, the lower PS of Ag^0^/CMC accounts for higher dispersion in the aqueous media and higher contact surface as compared with Cu^0^/CMC.

There exists a narrow ternary interdependence between the pH, zeta potential (ZP), and MNP/CMC dispersion in aqueous media [22]. A higher pH is assumed to induce the deprotonation of carboxymethyl group leading to a ZP increase that enhances the repulsion forces and material dispersion. This interdependence appears to govern the extent of the contact surface and exchange fluxes with the targeted microorganisms. 

The shapes of MNP in both Cu^0^/CMC and Ag^0^/CMC were similar to those already characterized by X-ray photoelectron spectroscopy (XPS) and transmission electron microscopy (SEM). The results revealed mostly pseudo-spherical 1–4 nm nanoparticles of zero-valent copper and silver, as previously reported with corresponding sizes in the same sequence and order of magnitude, with even 0.08–0.1 nm subnanoparticles for Ag^0^/CMC.

Their respective polydispersity indexes (PDI) of 0.96 ± 0.06 and 0.25 ± 0.02 (Table 1) reported here for Cu^0^/CMC and Ag^0^/CMC were higher than 0.05, which are regarded as the monodisperse standard [24]. 

### 3.2. Characterization of MNP/CMC–Carboxyl Interaction

**Figure 2:**
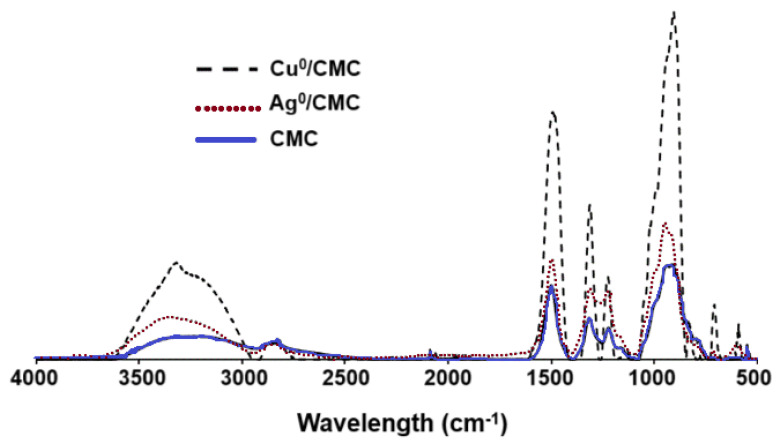
ATR−FTIR spectra of CMC, Cu^0^/CMC, and Ag^0^/CMC.

Deeper insights through attenuated total reflectance Fourier transform infrared (ATR-FTIR) analysis showed noticeable and specific changes in the shape and intensity of the large adsorption band in the 3500–3000 cm^−1^ region (Figure 2), with more intense and sharper bands for Cu^0^/CMC and Ag^0^/CMC in comparison with CMC. This suggests lesser hydrogen association (involving -OH groups of the CMC), which are hindered by the presence of MNP/CMC. Higher intensities, sharpness, and minor shifts of the -OH asymmetric stretching bands from 3255 cm^−1^ for CMC towards higher wavenumbers; i.e., 3343 cm^−1^ for Cu^0^/CMC and 3371 cm^−1^ for Ag^0^/CMC (Table 1) indicate a slight softening of the -OH bonds, which requires less energy for stretching vibration due to interaction with MNPs. This can be explained by the involvement of the hydroxyl groups of the CMC via the electron pairs of the oxygen atoms. More precisely, the slight band sharpening in the range of 3500–3000 cm^−1^ seems to be related to the rise in -O:MNP/CMC interaction at the expense of H-bridges. Thus, it appears that the hydroxyl groups are no longer involved in hydrogen binding but are involved in MNP/CMC stabilization. 

Differently, only minimal shifts were found for the band assigned to the -OH bending of the carboxyl group from 1411 cm^−1^ for CMC to 1409 cm−1 for both Cu^0^/CMC and Ag^0^/CMC. As expected, no MNP/CMC interaction with the hydroxyl groups of the saccharide cycles seems to occur, as much as the -OH bending band of polysaccharide remained almost unshifted (1323 for CMC, 1325 for Cu^0^/CMC, and 1326 cm^−1^ for Ag^0^/CMC). In accordance with our results, Raghavendra et al. [25] also observed peaks at this region from polysaccharide interactions.

MNP/CMC appear to also interact with the oxygen atoms of the C=O bonds belonging to the carboxyl, as supported by a noticeable shift of the C=O stretching in polysaccharides from 1591 cm^−1^ (CMC) to 1583 cm^−1^ (Cu^0^/CMC) and to 1588 cm^−1^ (Ag^0^/CMC).

Cu^0^NP and Ag^0^NP interaction with the polymeric chain seems to also involve the oxygen atoms of the saccharidic cycle, given the marked shift of the C-O bending and C-O-C stretching bands from 1021 cm^−1^ of CMC to 1015 cm^−1^ for Cu^0^/CMC and given the even more accentuated shift for Ag^0^/CMC (1110 cm^−1^). This band was also observed by Raghavendra et al. [25] as being provided by the carbohydrate CMC. Overall, the shifting of the peaks confirms the loading of metals by CMC.

### 3.3. Effect of Cu^0^/CMC and Ag^0^/CMC on IPEC-J2 Viability

To mimic the intestine environment of pigs, the porcine enterocyte cell line (IPEC-J2) was used as in vitro model. IPEC-J2 is a non-transformed and permanent columnar epithelial cell line isolated from neonatal piglet mid-jejunum [26]. The cells of this line express the receptor for fimbriae 4 (F4) [27]. These features make IPEC-J2 cells an interesting in vitro model to investigate the ways of F4 strains attachment on IPEC-J2 cells and to evaluate the antibacterial properties of MNP/CMC.

**Figure 3:**
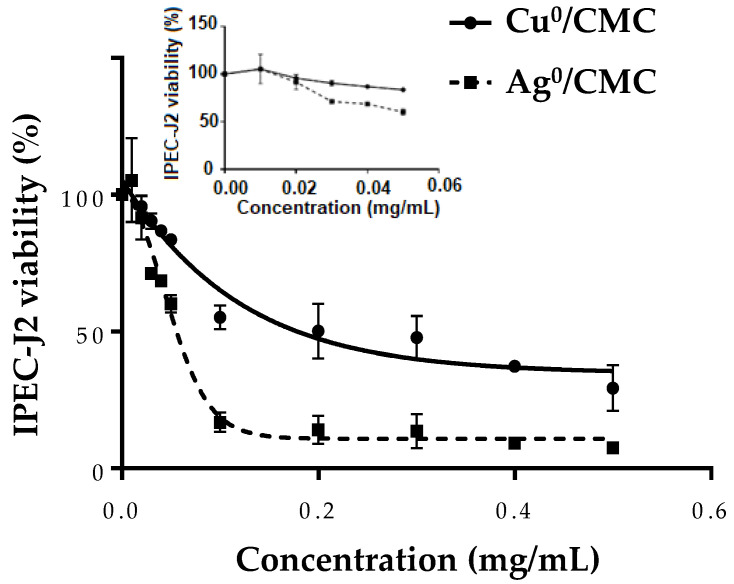
The effect of MNP/CMC at various concentrations (0–0.5 mg/mL; insert: 0–0.05 mg/mL) on the viability of non-infected IPEC-J2. After 24 h of treatment, the cytotoxicity of Cu^0^/CMC and Ag^0^/CMC was assayed by the MTT test. The closeup shows for a concentration of 0.05 mg/mL, there was a loss of viability of about 15% for Cu^0^/CMC and about 30% for Ag^0^/CMC. The data are the average of three different experiments.

The safety concentration range of Cu^0^/CMC or Ag^0^/CMC was evaluated via 3-(4,5-dimethylthiazol-2-yl)-2,5-diphenyl-2H-tetrazolium bromide (MTT) reduction by viable IPEC-J2. Non-infected IPEC-J2 cells were treated with Cu^0^/CMC or Ag^0^/CMC at various concentrations in the range of 0–0.50 mg/mL for 24 h (Figure 3). The minimum inhibitory concentration (MIC) of Cu^0^/CMC and of Ag^0^/CMC were close to each other: 0.027 ± 0.003 mg/mL and 0.021 ± 0.001 mg/mL, respectively (Figure 3 closeup and Table S1). Differently, the concentration that inhibits the growth of IPEC-J2 at half (IC_50_) was 0.201 ± 0.013 mg/mL for Cu^0^/CMC, approximately four times higher than that of 0.052 ± 0.003 mg/mL observed for Ag^0^/CMC (Table S1). A lower IC_50_ and MIC for Ag^0^/CMC compared with those of Cu^0^/CMC (Figure 3 and closeup) revealed that the cytotoxicity of Ag^0^/CMC for non-infected IPEC-J2 was higher compared with that of the Cu^0^/CMC counterpart. 

This favorable effect of Ag^0^/CMC toxicity may be related to the smaller particle sizes of both the material grains and of zero-valent metal; and to the increase in their surface-to-volume ratio. Both features make Ag^0^/CMC be regarded as suitable for acting on IPEC-J2. This hypothesis is in accordance with the impact of particle size mentioned by Danaei et al. [24].

### 3.4. Effect of MNP/CMC on ETEC:F4 Bacteria Viability

The biocidal activity of synthesized Cu^0^NP and Ag^0^NP hosted by CMC was evaluated on ETEC:F4 bacteria via the optical density at 600 nm. The ETEC:F4 EcL8559 strain was chosen in this study because it is the best characterized pathogen in swine postweaning diarrhea [28,29,30]. 

**Figure 4:**
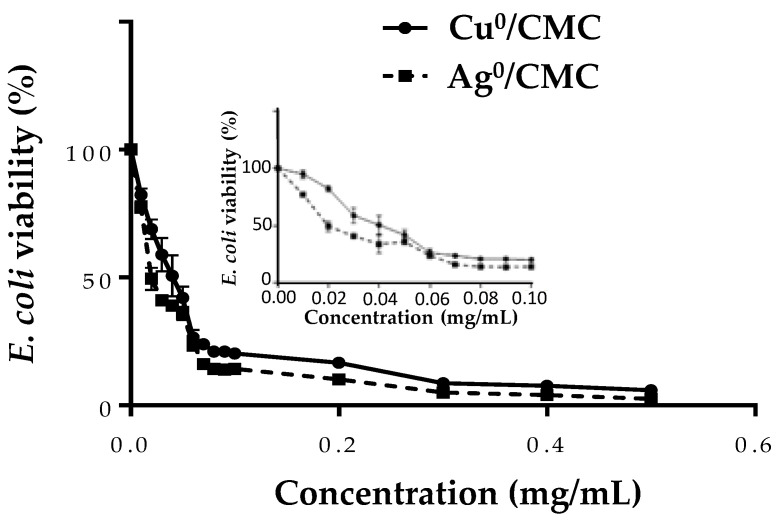
The bactericidal effect of MNP/CMC at various concentrations (0–0.5 mg/mL and insert: 0–0.1 mg/mL) on ETEC:F4, expressed as loss of bacteria survival. The bacteria (1 × 10^7^ CFU/mL) were treated with MNP/CMC at various concentrations for 24 h at 37 °C. The bacteria survival corresponds to the relative optical density at 600 nm of treated bacteria in solution compared with the untreated control (100%). The data are the average of three different experiments.

Bacteria (1 × 10^7^ CFU/mL) were treated with MNP/CMC at various concentrations (0 to 0.5 mg/mL) to investigate their bactericidal activity (Figure 4 close-up). The IC_50_ values were 0.046 ± 0.003 mg/mL for Cu^0^/CMC and 0.023 ± 0.002 mg/mL for Ag^0^/CMC, whereas the MIC values were similar: 0.010 ± 0.001 mg/mL for both agents (Figure 4, close-up, Table S1). The IC_50_ values showed that Ag^0^/CMC was the most bactericidal material for bacteria grown. The same tendency was also observed when the bacteria were treated with metal-loaded glycodendrimers [23], suggesting that the small distribution size of Ag^0^/CMC increased their toxicity, while Cu^0^/CMC with a larger particle size distribution, was less toxic for bacteria. The other main observation was that the IC_50_ of MNP/CMC on bacteria viability are approximatively 5–3 times lower than that observed on enterocyte cell line IPEC-J2, suggesting that bacteria are more sensitive than IPEC-J2. Considering the previous results, the concentrations used to investigate the bactericidal activity of MNP/CMC on IPEC-J2 was from 0 to 0.05 mg/mL, which represented a toxic concentration range for bacteria.

The antibacterial activity of MNP/CMC agents was also evaluated by a disk diffusion assay. The zone diffusion diameters on a 1 mg LB agar gel of some drugs such as kanamycin and fosfomycin, commonly used in veterinary or human health, were compared with those of the new MNP/CMC antibacterial agents (Table 2). As expected, no disk diffusion was observed with CMC treatment, reported as GRAS and used as a negative control. Compared with fosfomycin, the antibacterial activity of Cu^0^/CMC was similar, whereas Ag^0^/CMC was more bactericidal. In the same way, the antibacterial activity of Ag^0^/CMC was close (slightly lower) to that of the potent antibiotic kanamycin (Table 2). These results suggested that Ag^0^/CMC could be used as alternative to kanamycin against ETEC:F4 growth.

**Figure 5:**
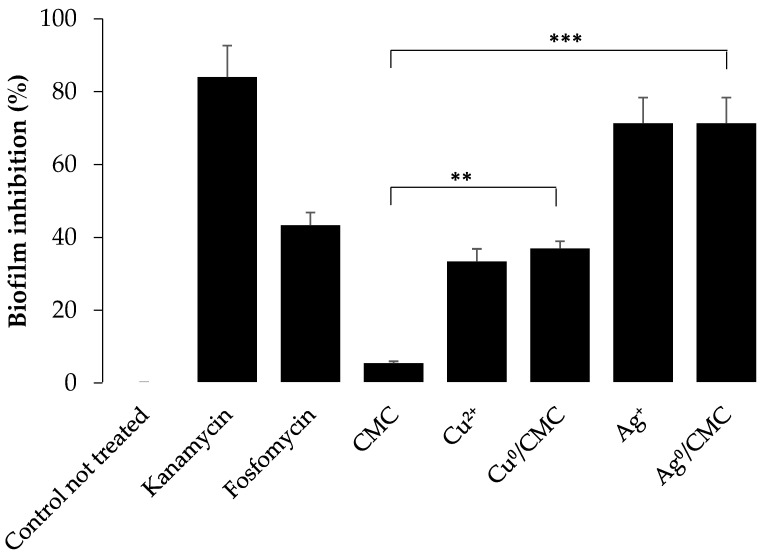
Inhibition of enterotoxigenic *E. coli* biofilm formation by treatment with various antibacterial agents for 48 h at 37 °C. Biofilm formation was quantified by a crystal violet assay (measured on microplate at λ = 570 nm). Readings were normalized in terms of percentage of inhibition with 100% biofilm formation as a negative control; untreated bacteria with 0% inhibition. (mean ± SD, n = 3 of three different experiments. ** *p* ≤ 0.05 and *** *p* ≤ 0.005).

In order to complete the knowledge on the antibacterial activity of MNP/CMC, the biofilm formation by ETEC:F4 bacteria was evaluated by a crystal violet assay (Figure 5 and Figure S1), based on the principle that dye intensity (at 570 nm) correlates with the abundance of the bacterial biofilm (Figure 5). As expected, the Cu^0^/CMC andAg^0^/CMC inhibited the formation of bacterial biofilm with the same tendency as the cation forms (Cu^2+^ and Ag^+^) in terms of the disk diffusion test (Table 2). The CMC matrix (control) showed no bactericidal toxicity (Table 2) and no inhibition of biofilm formation (Figure 5). In addition, we also found that kanamycin could be substituted by Ag^0^/CMC. The inhibition of the formation of biofilm related to MNP/CMC treatments confirm the higher bactericidal activity of Ag^0^/CMC as observed by El-Riz et al. (2024), with Ag^0^ and Cu^0^ being hosted by manno-dendrimers [23].

### 3.5. Cytotoxicity of MNP/CMC on the Proliferation of ETEC:F4 EcL8559 Strain on the Infected IPEC-J2

IPEC-J2 cells were infected with ETEC:F4 bacteria to mimic the infection in pigs. The viability of IPEC-J2 infected with ETEC:F4 was measured using the lactate dehydrogenase (LDH) assay (Promega, Canada) based on the release of intracellular enzyme LDH upon cell lysis. Different to the MTT method, based on the ability of living cells to cleave the tetrazolium ring and reduce the MTT to solid purple formazan-class dye, the LDH method evaluates the ability of MNP/CMC agents to disrupt cell membranes and release LDH into culture medium. LDH activity is an enzymatic reaction, where LDH oxidizes lactate to pyruvate, which reacts with yellow iodonitrotetrazolium chloride salt into a red formazan-class dye. Formazan dye is water soluble and can be readily detected by measuring the absorbance at 490 nm [31,32].

**Figure 6:**
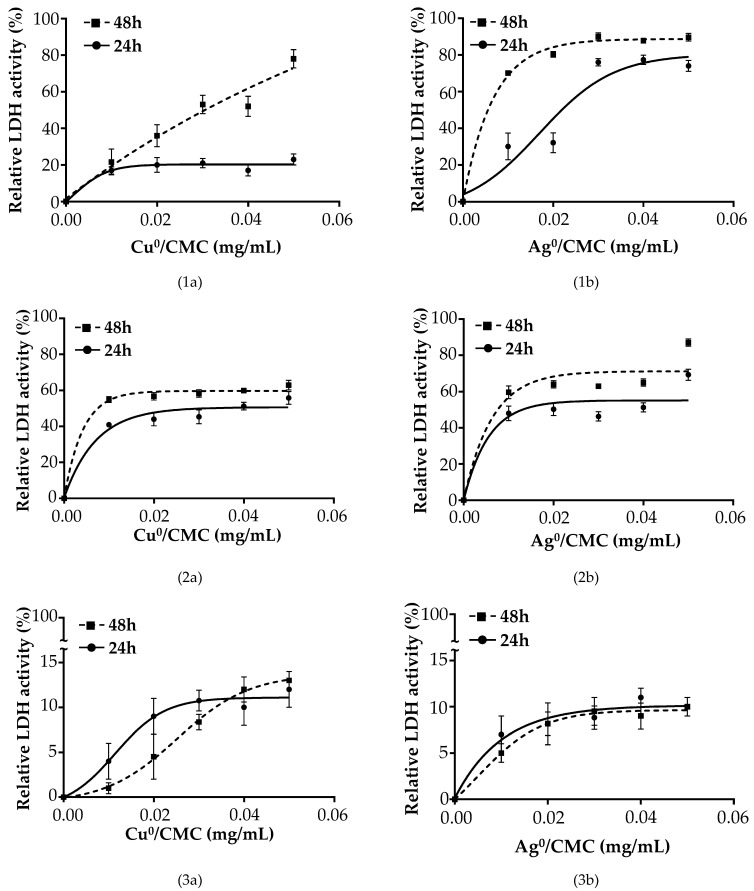
Biocidal effect of Cu^0^/CMC (**1a**–**3a**) and Ag^0^/CMC (**1b**–**3b**) at various concentrations on IPEC-J2 infected with ETEC:F4 in different conditions: (1) IPEC-J2 first infected with 1 × 10^7^ CFU/mL ETEC:F4 and then treated with Cu^0^/CMC (**1a**) or with Ag^0^/CMC (**1b**) for 24 h and 48 h; (2) IPEC-J2 first treated with Cu^0^/CMC (**2a**) or with Ag^0^/CMC (2b) for 24 h or 48 h and then infected with 1 × 10^7^ CFU/mL ETEC:F4 for 24 h; and (3) IPEC-J2 infected with the mixture obtained from 1 × 10^7^ CFU/mL ETEC:F4 bacteria previously treated for 24 h or 48 h with Cu^0^/CMC (**3a**) or with Ag^0^/CMC (**3b**) with MNP/CMC. In all three situations, IPEC-J2 cells were maintained at 37 °C, 5% CO_2_.

To understand the action mechanism of the MNP/CMC, three situations were envisaged. For situation 1: [{IPEC-J2 cells + ETEC:F4} + MNP/CMC] the results are presented in Figure 6(1a,1b); for situation 2: [{IPEC-J2 cells + MNP/CMC} + ETEC:F4] the results are presented in Figure 6(2a,2b); for situation 3: [IPEC-J2 + {ETEC:F4 + MNP/CMC }] the results are presented in Figure 6(3a,3b).

As previously mentioned, the IC_50_ of both MNP/CMC upon treatment of intestinal IPEC-J2 did not overlap with those obtained upon treatment of ETEC:F4 bacteria, and a concentration range of 0 to 0.05 mg/mL for both Cu^0^/CMC and Ag^0^/CMC was considered as suitable for investigating the beneficial effects of Cu^0^/CMC and of Ag^0^/CMC as anti-infectious agents on intestinal IPEC-J2 infected by the ETEC:F4 EcL8559 strain.

Figure 6 shows the viability profile of IPEC-J2 cells in the three situations (Situation 1 to 3). In Situations 1 and 2, the MNP/CMC treatment for 24 h led to a decrease in the LDH activity of 80 and 60% for Cu^0^/CMC (Figure 6(1a,2a)) and 25 and 30% for Ag^0^/CMC (Figure 6(1b,2b)), respectively, compared with the positive control (IPEC-J2 infected by ETEC:F4) under no protection by MNP/CMC, considered as the 100% release of LDH. 

The curve tendency representing the LDH activity of infected cells treated with MNP/CMC is similar at 24 h compared with 48 h of treatment, with an increase phase and a stationary phase, except for Cu^0^/CMC and Ag^0^/CMC having an almost constant increase up to 80% LDH activity at 48 h of treatment (Figure 6(1a,2a)).

In Situation 3, the IPEC-J2 infected with ETEC:F4 pretreated with MNP/CMC bactericidal agents showed a markedly low LDH release of about 19% for Cu^0^/CMC (Figure 6(3a)) and 17% for Ag^0^/CMC (Figure 6(3b)), meaning a good viability of infected IPEC-J2, when the ETEC:F4 bacteria was pretreated with MNP/CMC bactericidal agents. 

In all three situations, the IC_50_ of Cu^0^/CMC at treatment of up to 48 h on IPEC-J2 cells was 0.102 ± 0.017 mg/mL, whereas the IC_50_ values of Ag^0^/CMC-treated IPEC-J2 cells were 0.06 ± 0.005 mg/mL at 24 h and 0.038 ± 0.004 mg/mL at 48 h of treatment with ETEC:F4 (Table S1a), suggesting much higher bactericidal properties for Ag^0^/CMC but good tolerability by the IPEC-J2 at the selected range concentrations. 

For both Ag°/CMC and Cu°/CMC, there is a higher toxicity on *E. coli* bacteria than on intestinal IPEC-J2 cells. This is a good result, showing high bactericidal activity and low cytotoxicity on intestinal cells and tissues. Furthermore, on the ETEC:F4 strain, the Ag°/CMC presented a higher bactericidal activity than that of Cu°/CMC. A similar trend was found on intestinal IPEC-J2 cells, with a higher cyto-toxicity of Ag°/CMC than that of Cu°/CMC, with the note that for both agents, the effect was moderate to low when compared with the effect on ETEC:F4 microorganisms. 

The differences in terms of bactericidal activity may be explained by the key role of the particle size (PS) of the metal-loaded biopolymer, given that Ag°/CMC display a lower average diameter (210 ± 11 nm) as compared with Cu°/CMC (369 ± 23 nm), as shown in Figure 1, and a lower polydispersity index for Ag°/CMC (0.25 + 0.02) than for Cu°/CMC (0.96 + 0.06). This accounts for a higher contact surface with the infected aqueous medium.

These antibacterial properties induced by zero-valent metal nanoparticles are supported by control tests. The latter clearly demonstrated that CMC alone had no effect on bacteria, as no antibacterial activity was detected. Here, CMC is assumed to promote an optimal metal retention strength for simultaneously maximum metal stabilization and contact surface through minimum material particle size.

The IC_50_ of MNP/CMC for non-infected IPEC-J2 cells (Table S1, Figure 4) was approximately two times higher for Ag^0^NP/CMC or four times higher for Cu^0^NP/CMC than that in cells infected with ETEC:F4 bacteria (Table S2, Figure 6), meaning a low toxicity for the epithelial cells of intestinal tissue. A possible explanation of this result could be a high ability of MNP/CMC to penetrate bacteria [33] but not eukaryote cells. Thus, significant damage was supposed at the MNPs uptake by ETEC:F4 [34]. In our case, the damage was more pronounced with Ag^0^NPs from Ag^0^/CMC than with Cu^0^NPs from Cu^0^/CMC with a larger size (Table 1), emphasizing that the size of the particle is an important parameter related to their cell cytotoxicity [35,36].

The infective effect of bacteria was time-related (Figure S2a). Meanwhile, the non-infected IPEC-J2 and ETEC:F4 were treated with CMC polymer alone at various concentrations (0–0.5 mg/mL) used as the negative control, and non-significant effects (*p* > 0.05) on both non-infected IPEC-J2 and on ETEC:F4 viability were found (Figure S2b). In opposition to different anaerobic Gram-positive and Gram-negative gut bacteria such as *Ruminococcus albus* and *Bacteroides cellulosolvens* (found in the lumen of major ruminants) that are able to degrade cellulose, *E. coli* is not able to metabolize cellulose [37,38]. This can explain why treatment with CMC alone did not have any effect on the proliferation or on ETEC:F4 viability.

The MTT viability assay on infected cells showed an increased formazan formation compared with non-infected cells, considered as 100% viability. It was reported that ETEC:F4 reduces MTT [39] and the increase in absorbency above 100% viability could be explained by the interference of the ETEC:F4 signal to that of IPEC-J2. In contrast, no significant variation of LDH activity was observed in relation to the presence of bacteria, supporting the previous statement that the MTT kit could be also used to assay bacteria viability [40]. The increase in formazan crystal formation could be due to the cell surface-attached bacteria in accordance with [41], which showed that ETEC:F4 expressing F4 can bind IPEC-J2 cells and release enterotoxins. Consequently, the interfering increased MTT reduction to formazan by IPEC-J2 in the presence of bacteria and justified the choice for the LDH assay to evaluate the viability of IPEC-J2 infected with ETEC:F4.

Fosfomycin is an antibiotic used in pig farms to inhibit *E. coli* infections and proliferation. It was used in this study to compare the bactericidal potency of Cu^0^/CMC and of Ag^0^/CMC with that of currently used antibiotics. The bactericidal activity of fosfomycin observed against ETEC: F4 (Table 2) was lower than that of Ag^0^/CMC. Compared with kanamycin, the zero-valent Ag^0^/CMC nanoparticles inhibited ETEC:F4 EcL8559 strain proliferation (Table 2) with a bactericidal potency of the same order (slightly lower) and also efficiently inhibited bacterial biofilm formation (Figure 6). Its strong bactericidal potential on *E. coli*, as shown by its IC_50_, approximately four times lower than that of Cu^0^/CMC (Figure 5), was observed with the crystal violet test used for the quantification of bacteria biofilm formation (Figure 6). The bactericidal properties of Cu^0^/CMC (NPs) and Ag^0^/CMC (NPs), with a higher antibacterial potency of Ag^0^/CMC (NPs) related to their smaller size, were reported by Noori et al. [22,42,43] in the case of non-pathogenic *E coli* DH5α, showing the role of MNP/CMC dispersion in the CMC hosting matrix. To reduce the effect of enterotoxigenic *E. Coli* contaminations in swine industries, the antibacterial effects of MNP/CMC agents could be proposed for further investigations and developments as alternative agents against pathogenic bacteria-related infections. In addition, to assess the therapeutic potential and safety of zero-valent MNP/CMC, the next part of this project will be aimed at in vivo studies, using weaning piglets as an animal model.

## 4. Conclusions

Nanomaterials consisting of zero-valent Cu^0^ and Ag^0^ hosted by carboxymethyl cellulose (CMC) exerted bactericidal activities with a stronger antibacterial action of Ag^0^/CMC compared with Cu^0^/CMC. It was found that intestinal porcine enterocytes IPEC-J2 represent a good model to investigate ETEC:F4 bacteria infectivity and cytotoxicity and that Ag^0^/CMC and Cu^0^/CMC prevented the infection of intestinal cells by enterotoxigenic *E. coli* (ETEC:F4). 

The concentrations of MNP/CMC must be tailored to selectively target the death of ETEC:F4 bacteria and not that of IPEC-J2. Higher bactericidal effectiveness was shown by the smaller particle size of MNP/CMC and their lower polydispersity index. In addition, Cu^0^/CMC and Ag^0^/CMC have antibacterial activities that fit well with the data obtained with the currently used antibiotics kanamycin and fosfomycin. Further studies will be conducted with the aim of determining the mechanisms of the antibacterial activity of Cu^0^/CMC and of Ag^0^/CMC and investigating their antibacterial effect *in vivo* on post-weaned piglets with ETEC:F4 infection symptoms.

## Figures and Tables

**Table 1:** Some properties of CMC and the synthesized Cu0/CMC and Ag0/CMC samples.

Characteristics	Measured Parameters *	CMC	Cu^0^/CMC	Ag^0^/CMC
Particle size and dispersion	Z average (nm)		367 ± 39	218 ± 17
	Average diameter (nm)		369 ± 23	210 ± 11
	PDI		0.96 ± 0.06	0.25 ± 0.02
Main IR vibration bands (cm^−1^)	O-H vibration	3255	3343	3371
	C-H stretching of -CH_2_ group	2872	2872	2882
	C=O stretching of polysaccharides	1591	1583	1588
	O-H bending carboxylic group	1411	1409	1409
	O-H bending of polysaccharide chains	1323	1325	1326
	C-O bending and C-O-C stretching	1021	1015	1110

* Biophysical properties of Cu^0^/CMC and Ag^0^/CMC included the particle size and infrared (IR). Data of samples were acquired during the dynamic light scattering (DLS) processing and via attenuated total reflectance Fourier transform infrared (ATR–FTIR) experiments.

**Table 2:** Antibacterial activity of MNP/CMC in comparison with that of current antibiotics used for human and porcine treatments. The diameters of inhibition zones were measured at 1 mg of antibacterial agents.

Antibacterial Agents		Inhibition Diameter (cm)
Human-used antibacterial agent	Kanamycin	2.6 ± 0.33
Porcine-used antibacterial agent	Fosfomycin	1.2 ± 0.21
	CMC	-
Metal nanoparticles	Cu^0^/CMC	1.0 ± 0.1
	Ag^0^/CMC	2.2 ± 0.3
Metal ions *	Cu^2+^	1.2 ± 0.1
	Ag^+^	2.4 ± 0.2

* It is worth noting that even the metal ions presented a bactericidal effect; the formulation as zero-valent metals hosted by CMC as MNP/CMC is essential in order to prevent toxicity and undesired interactions with various elements upon contact with tissues and cells. For instance, free Ag^+^ in contact with physiological materials as NaCl or KCl may produce rapidly solid AgCl salt on site. The Cu^2+^ and Ag^+^ ions are both from commercial products of copper II acetate [Cu(CH_3_COO)_2_] and silver nitrate (AgNO_3_), respectively.

## Data Availability

Data are contained within the article and Supplementary Materials.

## Supplementary Materials

The following supporting information can be downloaded at: https://www.mdpi.com/article/10.3390/microorganisms12102026/s1, Table S1: Minimum inhibitory concentration (MIC) and half inhibitory concentration (IC_50_) of MNP/CMC on IPEC-J2, uninfected or infected by ETEC:F4 bacteria. Table S2: Inhibitory concentration (IC_50_ in mg/mL). Figure S1: Quantification of biofilm formation by a crystal violet assay at 570 nm. Figure S2: IPEC-J2 infected by ETEC:F4 bacteria.

## List of Abbreviations

Ag^0^/CMC: nanoparticles of zero-valent silver loaded on CMC; CFU: colony-forming units; CMC: carboxymethyl cellulose; Cu^0^/CMC: nanoparticles of zero-valent copper loaded on CMC; DS: degree of substitution of CMC; ETEC: enterotoxigenic *Escherichia coli*; F4: fimbriae 4; ETEC:F4: enterotoxigenic *Escherichia coli* producing *fimbriae* 4; IC_50_: half maximal inhibitory concentration; LB: Luria broth or Luria–Bertani medium; M: zero-valent metal (Cu^0^, Ag^0^); MIC: minimal inhibitory concentration; MOI: multiplicity of infection; MNPs: metal nanoparticles; MNPs/CMC: Nanoparticles of zero-valent metal loaded on CMC; NPs: nanoparticles; PDI: polydispersity index.
