# Supplementary material for: Silver and Copper Nanoparticles Hosted by Carboxymethyl Cellulose Reduce the Infective Effects of Enterotoxigenic Escherichia coli:F4 on Porcine Intestinal Enterocyte IPEC-J2
Source: Microorganisms. 2024 Oct 7;12(10):2026. doi: 10.3390/microorganisms12102026 (PMC11509932; doi:10.3390/microorganisms12102026)
Supplement: Supplementary file 1 [file microorganisms-12-02026-s001.zip › microorganisms-3194715-supplementary.pdf]

Supplement information

Silver and copper nanoparticles hosted by carboxymethyl cellulose reduce the infective effects of enterotoxigenic *Escherichia coli* F4 on porcine intestinal enterocyte IPEC-J2

Armelle Tchoumi Neree*, Farzaneh Noori, Abdelkrim Azzouz, Marcio Costa, John Morris Fairbrother, Mircea Alexandru Mateescu *, and Younes Chorfi

* Correspondence: armelle.tchoumi.neree@umontreal.ca (ATN); Mateescu.m-alexandru@uqam.ca (MAM); Tel: +1450 773-8521, #13439 (ATN); +1514 986-4319 (MAM)

Table S1. Summary of MIC and IC_{50} of CMC-hosted MNP/CMC on IPEC-J2 cells uninfected or infected by ETEC EcL8559 bacteria strain.

IPEC-J2 cells	Measured parameters (mg/mL)	Cu^0/CMC	Ag^0/CMC
	MIC	0.027±0.003	0.021±0.001
Uninfected cells	1/2 IC_{50}	0.052±0.004	0.024±0.002
	IC_{50}	0.201±0.013	0.052±0.003
	MIC	0.010±0.001	0.010±0.001
Infected cells	1/2 IC_{50}	0.025±0.002	0.012±0.001
	IC_{50}	0.046±0.003	0.023±0.002

The IPEC-J2 cells were treated with Cu^0/CMC or Ag^0/CMC for 24 h. For IPEC-J2 cells infected with ETEC:F4 EcL8559, the infection time was 16 h at 37 °C.

Table S2. Summary of IC_{50} (mg/mL) obtained in situations 1 to 3.

	M/CMC(NPs) treatment	IC_{50} Time incubation (h) 24	48
Situation 1: (IPEC-J2 + bacteria) + MNP/CMC	**Cu^0/CMC**	ND	0.102 ± 0.0017
	**Ag^0/CMC**	0.060 ± 0.005	0.038 ± 0.004
Situation 2: (IPEC-J2 + MNP/CMC) + Bacteria	**Cu^0/CMC**	ND	ND
	**Ag^0/CMC**	ND	ND
Situation 3: IPEC-J2 + (bacteria + MNP/CMC)	**Cu^0/CMC**	0.098 ± 0.014	ND
	**Ag^0/CMC**	0.049 ± 0.005	0.05 ± 0.003

The IC_{50} of Cu^0/CMC and of Ag^0/CMC was obtained after 24 and 48 h treatments at 37 °C, 5% CO_2. In situation 1 the IPEC-J2 cells were infected with ETEC:F4 EcL8559 bacteria and then treated with MNP/CMC. In situation 2, the IPEC-J2 cells were treated with MNP/CMC then the infection process was occur with ETEC:F4 EcL 8559 bacteria In situation 3, the solution resulting from the treatment of ETEC:F4 EcL8559 bacteria by MNP/CMC was used to evaluate the behavior of IPEC-J2 cells.

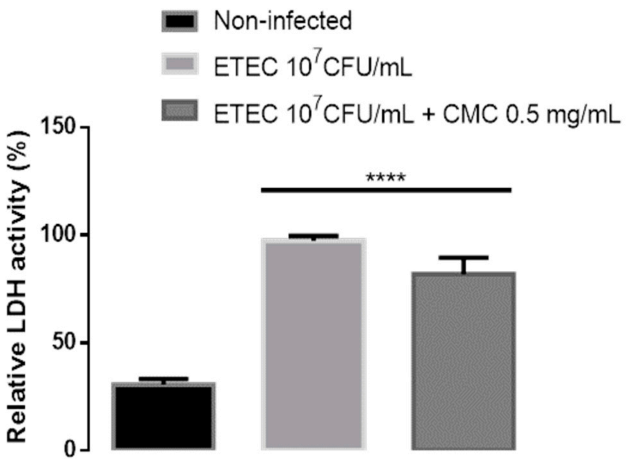

(**a**) (**b**)

Figure S1. IPEC-J2 cells infected by ETEC:F4 EcL8559 bacteria. a-The IPEC-J2 cells infected or not were incubated at 37ºC, 5% CO_2 up to 72 h with or without ETEC 10^7 CFU/mL. b-The IPEC-J2 cells previously infected with ETEC:F4 EcL8559 bacteria $1x10^7$ CFU/mL for 24 h were treated with CMC 0.5 mg/mL for 24 h. Statistical analysis was realized with Anova one way ****p≤0.0001.

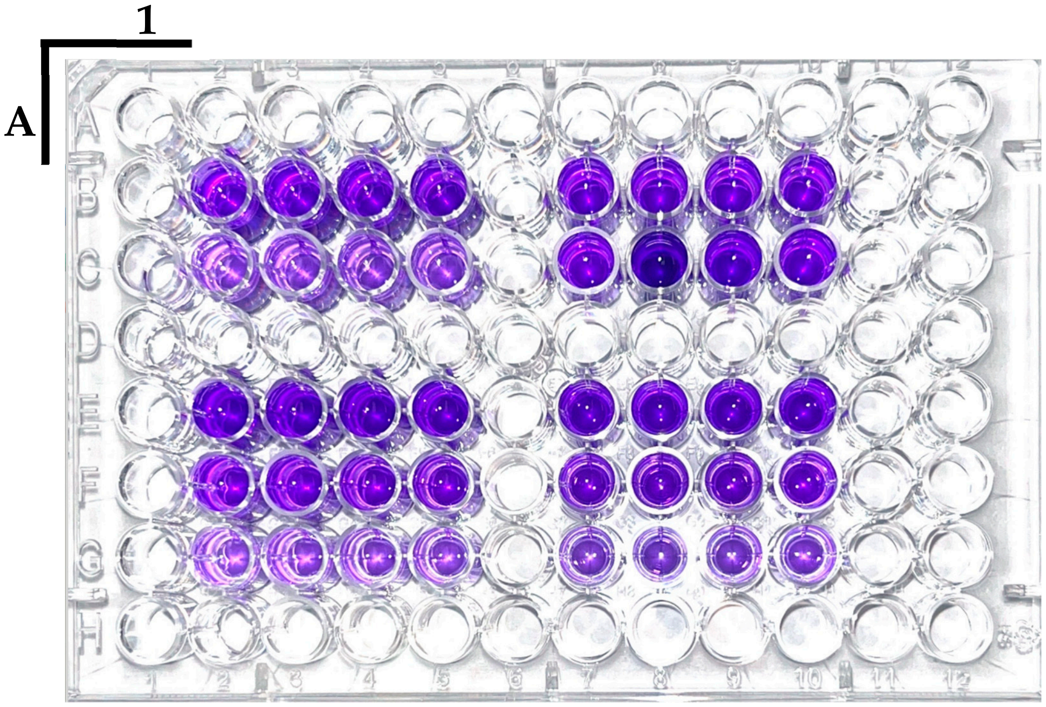

Figure S2. Biofilm formation was evaluated by crystal blue assay. The color intensity was related to biofilm quantity. B2-5 and B7-10: CMC; C2-5: kanamycin, B7-10: fosfomycin; E2-5 and E7-10: Control not treated; F2-5: Cu^{2+}; F7-10 : Cu^0/CMC; G2-5: Ag^+ and G7-10: Ag^0/CMC.
